# Missing Regulation Between Genetic Association and Transcriptional Abundance for Hypercholesterolemia Genes

**DOI:** 10.3390/genes16010084

**Published:** 2025-01-15

**Authors:** Aaron Hakim, Noah J. Connally, Gavin R. Schnitzler, Michael H. Cho, Z. Gordon Jiang, Shamil R. Sunyaev, Rajat M. Gupta

**Affiliations:** 1 Division of Genetics and Cardiovascular Medicine, Department of Medicine, Brigham and Women’s Hospital, Boston, MA 02115, USA; ahakim@bidmc.harvard.edu (A.H.); gavin@broadinstitute.org (G.R.S.); 2 Division of Gastroenterology and Hepatology, Beth Israel Deaconess Medical Center, Boston, MA 02215, USA; zgjiang@bidmc.harvard.edu; 3 Channing Division of Network Medicine, Brigham and Women’s Hospital, Boston, MA 02215, USA; remhc@channing.harvard.edu; 4 Broad Institute of MIT and Harvard, Cambridge, MA 02142, USA; noahconnally@g.harvard.edu (N.J.C.); ssunyaev@hms.harvard.edu (S.R.S.); 5 Department of Biomedical Informatics, Harvard Medical School, Boston, MA 02215, USA

**Keywords:** colocalization, eQTL, GWAS, gene regulation, genomics, LDL cholesterol, hypercholesterolemia

## Abstract

**Background:** Low-density lipoprotein cholesterol (LDL-C) is a well-established risk factor for cardiovascular disease, and it plays a causal role in the development of atherosclerosis. Genome-wide association studies (GWASs) have successfully identified hundreds of genetic variants associated with LDL-C. Most of these risk loci fall in non-coding regions of the genome, and it is unclear how these non-coding variants affect circulating lipid levels. One hypothesis is that genetically mediated variation in transcript abundance, detected via the analysis of expressed quantitative trait loci (eQTLs), is key to the biologic function of causal variants. Here, we investigate the hypothesis that non-coding GWAS risk variants affect the homeostatic expression of a nearby putatively causal gene for serum LDL-C levels. **Methods:** We establish a set of twenty-one expert-curated and validated genes implicated in hypercholesterolemia via dose-dependent pharmacologic modulation in human adults, for which the relevant tissue type has been established. We show that the expression of these LDL-C genes is impacted by eQTLs in relevant tissues and that there are significant genomic-risk loci in LDL-GWAS near these causal genes. We evaluate, using statistical colocalization, whether a single variant or set of variants in each genetic locus is responsible for the GWAS and eQTL signals. **Results:** Genome-wide association study results for serum LDL-C levels demonstrate that the 402 identified genomic-risk loci for LDL-C are highly enriched for known causal genes for LDL-C (OR 527, 95% CI 126–5376, *p* < 2.2 × 10^−16^). However, we find limited evidence for colocalization between GWAS signals near validated hypercholesterolemia genes and eQTLs in relevant tissues (colocalization rate of 26% at a locus-level colocalization probability > 50%). **Conclusions:** Our results highlight the complexity of genetic regulatory effects for causal hypercholesterolemia genes; we suggest that context-responsive eQTLs may explain the effects of non-coding GWAS hits that do not overlap with standard eQTLs.

## 1. Introduction

Cardiovascular disease is the leading cause of death in the developed world [1]. Low-density lipoprotein cholesterol (LDL-C) is a well-established risk factor for cardiovascular disease, and it plays a causal role in the development of atherosclerosis [2]. Clinical trials have shown, and European guidelines recommend, that individuals with an elevated risk of cardiovascular disease should target LDL levels below 55 mg/dL, with a level of 100 mg/dL considered optimal in the general population [2]. There are currently eight different classes of drugs approved by the Food and Drug Administration to lower cholesterol levels, which generally act to remove LDL-C from circulation and into liver cells [3]. Genome-wide association studies (GWASs) have successfully identified hundreds of genetic variants associated with either increased or decreased serum LDL-C [4]. Over 90% of these variants fall in non-coding regions of the genome, and it is unclear how these non-coding GWAS variants affect LDL-C. One hypothesis is that variants influence proximal gene expression. Supporting this, lipid GWAS loci are enriched in regulatory chromatin states, including enhancers and promoters, in hepatocytes [5]. Additionally, extensive efforts have cataloged thousands of expression quantitative trait loci (eQTLs), where the genotype of a single nucleotide polymorphism (SNP) correlates with the steady-state homeostatic expression of a nearby gene assayed in post mortem tissue samples from healthy subjects [6]. Some LDL-associated variants have been shown to overlap with the strongest eQTL for the region [4,5]. For example, the single non-coding DNA variant rs12740374 is associated with LDL-C in genome-wide association studies, and it also alters the expression of the *SORT1* gene in human liver samples [7]. Collectively, these observations suggest that GWAS hits for LDL-C could alter the transcriptional abundance of nearby genes. 

However, there is growing evidence that genetic variants associated with complex traits have minimal effects on homeostatic gene expression. Colocalization approaches have been developed to identify and quantify the overlap between eQTL and GWAS hits while ruling out genetic linkage as an explanation for overlap [8,9]. These approaches, which test the hypothesis that a shared variant or variants causally impact both the disease and gene expression, show that less than 50% of GWAS hits colocalize with eQTLs for complex traits [10,11]. Additionally, only a modest 11% of complex heritability has been shown to be mediated via gene expression in tissues from the Genotype-Tissue Expression project (GTEx) [12]. GWAS hits also lie at relatively long distances from transcription start sites, whereas eQTLs are tightly clustered near the transcriptional start site [13]. These and other observations suggest a missing link between genetic association and regulatory function [14,15].

The observation that most non-coding GWAS hits do not colocalize with eQTLs does not disprove the hypothesis that important trait-associated variants influence proximal gene regulation, as the causal gene for GWAS hits is usually unknown, and eQTLs may only be significant in a single tissue, in a single cell type, or during development. Additionally, it should be expected that not all GWAS peaks will colocalize with eQTLs, as most genes do not show haploinsufficient phenotypes; for example, in Drosophila, only 50 genes show haploinsufficient phenotypes [16,17]. Most genes are expressed in considerable excess [17], and in humans, most heterozygotes bearing a loss of function mutation in one allele are phenotypically normal. Therefore, an ideal test of the eQTL and GWAS colocalization hypothesis would be to examine a subset of true positive cases, in which the gene driving the trait is known, the biology is well understood, the relevant tissue type is identified, and the gene’s expression is not associated with a specific developmental stage, and for which the trait is known to display dosage sensitivity with respect to the gene’s expression or protein activity. Consequently, in this study, we investigate the hypothesis that non-coding GWAS risk variants affect the expression of a nearby putatively causal gene for serum LDL-C levels. Serum LDL-C is a complex trait for which multiple causal genes have been identified, characterized, and therapeutically targeted in adults. Serum LDL-C also displays dosage sensitivity with respect to target modulation. Using publicly available drug development databases, we established a set of 21 expert-curated and validated genes implicated in hypercholesterolemia. We demonstrate that GWAS hits for LDL-C are highly enriched for these known causal genes affecting serum LDL-C. We show that these hypercholesterolemia disease genes are influenced by baseline eQTLs in relevant tissues. However, we find limited evidence for colocalization between GWAS signals in validated hypercholesterolemia disease genes and homeostatic eQTLs in relevant tissues. This “colocalization gap” calls into question the hypothesis that genetic variants contribute to complex traits by altering homeostatic gene expression, and it motivates the development of experimental approaches to connect non-coding GWAS genetic variants to gene expression and cardiovascular disease risk.

## 2. Methods

### 2.1. Identifying Putatively Causal Genes Influencing LDL-C

Biomedtracker Version 4.180.0 (Citeline Commercial), an independent database and research service that tracks and analyzes developmental drugs through the FDA approval process, was used to curate a list of all pharmacologic agents and targets tested in Phase 1, Phase 2, and/or Phase 3 clinical trials using the search terms “hyperlipidemia”, “dyslipidemia”, “hypercholesterolemia”, “lipemia”, “hyperlipoproteinemia”, “lipids”, and “atherosclerosis” [18]. A separate search was also performed using these keywords on www.clinicaltrials.gov, accessed on 5 February 2024 [19]. A manual review of all drug and drug targets with an accompanying literature review by three internal medicine board-certified physicians was used to identify a subset of 21 expert-curated genes for which dose-dependent pharmacologic inhibition is known to impact serum LDL-C levels in adult humans. 

### 2.2. LDL-C GWAS Meta-Analysis and Enrichment for Positive Control LDL-C Genes

Summary statistics from the largest meta-analysis of European ancestry-specific GWASs of LDL-C (*n* = 1,320,016) were uploaded to the FUnctional Mapping and Annotation of GWAS tool (FUMA; version 1.5.6) [4,20]. We used the 1000G Phase3 EUR population as the reference population for calculating linkage disequilibrium (LD). We identified independent genomic-risk loci defined by a genome-wide significance value of *p* < 5 × 10^−8^, cataloged genes within 50 kb of the peak associated SNPs, and assessed which of our 21 positive control genes were mapped as GWAS loci. We used Fisher’s exact test to estimate the enrichment for positive LDL-C control genes among the closest genes to the genome-wide significant genomic-risk loci. 

### 2.3. UK Biobank LDL-C GWAS Adjusted for Coding Variation and eQTL Colocalization

We used previously published UK Biobank LDL-C GWAS summary statistics in European-ancestry subjects adjusted for coding variants [14]. Briefly, coding variants were identified for genes with strong expression in liver, whole blood, subcutaneous adipose, and visceral and omental adipose tissue (>50% of maximum expression across tissue) and that fell within the coding sequence of >75% of splice isoforms in that tissue [21]. These variants were used as covariates for the GWAS using plink 2.0, also adjusting for age, sex, body mass index, and the first 10 principal components of ancestry. We used data from the GTEx project, which profiled bulk postmortem adult tissue samples [10], to assess which of our set of 21 genes had an eQTL in at least one relevant tissue (adipose, liver, small intestine, and whole blood). We then implemented Baysian colocalization analysis as implemented in coloc version 4 [8] using the coding variant-adjusted LDL-C GWAS summary statistics from UK Biobank and GTEx v7 summary statistics (available at the time of coloc version 4) for relevant tissues. We also applied MASH (multi-variate adaptive shrinkage method) to non-brain GTEx tissues using the mash R-package version 0.2.79 [22]; by determining patterns of similarity across tissue types, MASH updates the GTEx summary statistics of each individual tissue. Coloc was rerun using the MASH-adjusted values for the same tissues as before. We defined significant colocalization using two posterior probability threshold cutoffs, h4 > 0.9 and h4 > 0.5, for the GWAS hit and eQTL being present and due to the same SNP. LocusCompare and eQTpLot were used to visualize colocalization between eQTL and GWAS signals [23,24].

### 2.4. FastEnloc and Colocalization with Fine-Mapped LDL-C GWAS

We next employed fast enrichment estimation-aided colocalization analysis (fastEnloc) [25,26], which uses a novel fine mapping-based strategy, to conduct assessments for colocalized SNPs. We ran fastEnloc using GTEx v8 and three different statistically fine-mapped GWAS results for LDL-C [4,27,28] from causalDB [29], including the largest meta-analysis of European ancestry-specific GWAS of LDL-C, and we applied a posterior probability cutoff of >0.5 and >0.9 to assess for colocalization. 

## 3. Results

We assembled a list of 21 causal genes for serum LDL-C by pharmacologic evidence in human adults; 19 of these genes, when inhibited, result in dose-dependent, clinically meaningful, statistically significant decreases in circulating LDL-C, and 2 of these genes, when inhibited, result in increases in LDL-C (Table 1). For these drugs and drug targets, the biology is well understood, the relevant tissue type has been identified, gene expression is not associated with a specific developmental stage, and there is dosage sensitivity with respect to target engagement for serum LDL-C levels.

**Figure 1:**
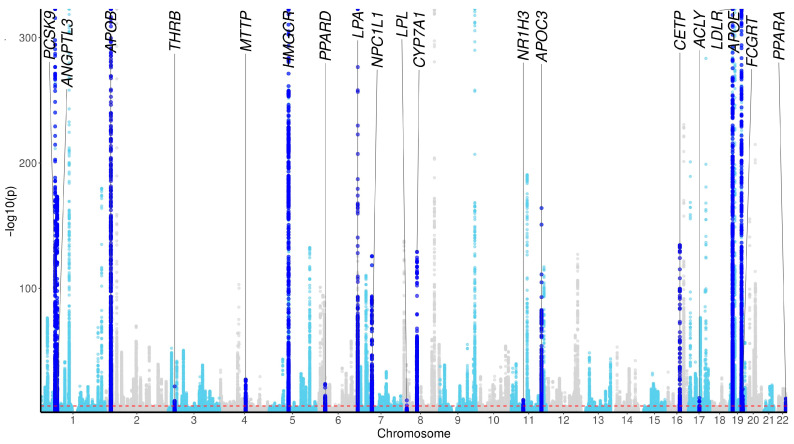
Manhattan plot illustrating that LDL-C GWAS loci are enriched for pharmacologically validated hypercholesterolemia targets; plot reflects meta-analysis of European ancestry-specific GWAS of LDL-C (*n* = 1,320,016) identifying 402 genomic-risk loci. Of our set of 21 validated hypercholesterolemia disease genes, 19 genes are suggestively implicated by GWAS (closest gene to a significant genomic locus). Dark blue peaks reference the closest gene to a significant genomic locus and also a known validated hypercholesterolemia disease gene.

We analyzed summary statistics from the largest meta-analysis of European ancestry-specific GWAS of LDL-C (*n* = 1,320,016) [4] to assess which of our 21 expert-curated and validated hypercholesterolemia genes were mapped as GWAS loci. In total, there were 402 significant LDL-C genomic loci. Of our set of 21 pharmacologically validated hypercholesterolemia genes, 19 genes were the closest gene to a significant LDL-C genomic locus, representing 527-fold enrichment compared to what is expected by chance (95% confidence interval: 126 to 5376; Fischer’s exact *p* < 2.2 × 10^−16^) (Table 2 and Table 3, Figure 1). We investigated how many of the 21 genes had an eQTL in at least one relevant tissue using GTEx, which is well powered for eQTL discovery in bulk tissue [10]. Under the stringent false discovery rate (FDR) statistics of the GTEx project, which accounts for testing every gene in every tissue, 19/21 (90.5%) of our genes had an eQTL in a relevant tissue type. Controlling for the number of tests we conducted, we found that all genes had an eQTL in at least one relevant tissue (Table 2).

**Figure 2:**
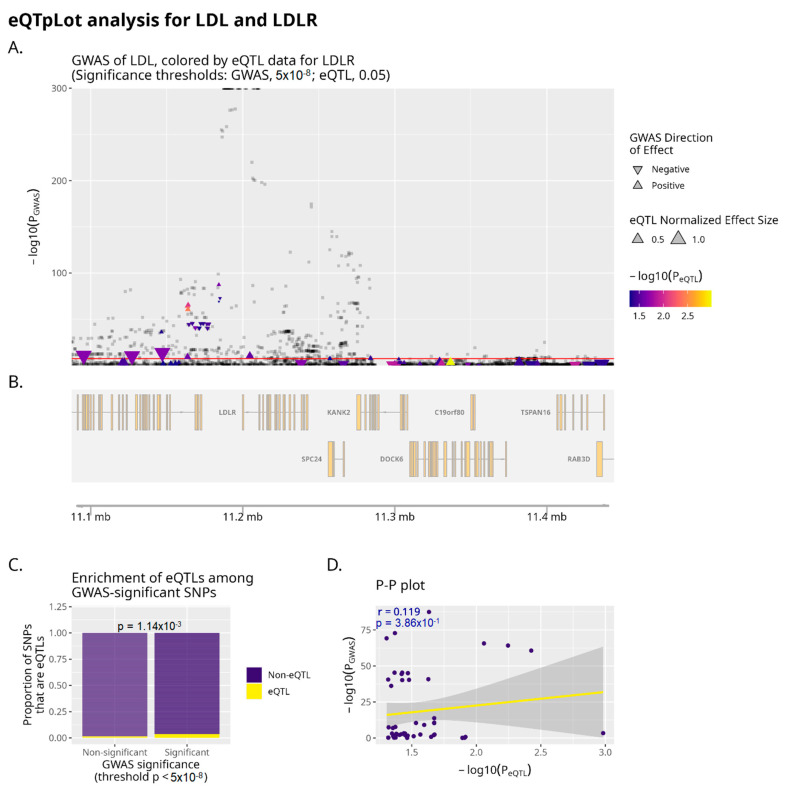
Lack of eQTL and GWAS colocalization at the *LDLR* locus in liver. Plot reflects UK Biobank summary statistics and GTEx v7 liver tissue. In (**A**), all variants significantly associated with LDL cholesterol are plotted above the horizontal red line. Variants associated with expression level of LDLR are indicated by coloration in orange or yellow. This plot demonstrates that variants significantly associated with LDL cholesterol do not colocalize with variants associated with LDLR expression levels. (**B**) indicates genomic position and genes in region. (**C**) shows only modest enrichment (*p* = 1.14 × 10^−3^ by Fisher’s exact test) for *LDLR* eQTLs among GWAS-significant variants. (**D**) illustrates a lack of evidence for correlation between P_GWAS_ and P_eQTL_ for the analyzed variants, with a Pearson correlation coefficient of 0.119, and a *p*-value of correlation of 3.86 × 10^−1^. Taken together, this analysis provides poor evidence for colocalization between variants associated with LDL cholesterol levels, and variants associated with LDLR expression levels.

We assessed colocalization between LDL-C GWAS signals and eQTLs in relevant tissues. First, we applied coloc version 4 using LDL-C GWAS results from UK Biobank adjusted for coding variants and GTEx v7 summary statistics. There were a total of 386 genes with a significant eQTL and a significant GWAS hit in the same region (h3 + h4 > 0.5) in at least 1 tissue type. In 113 instances, both the GWAS hit and eQTL were present and due to the same SNP, reflecting a 29% rate of colocalization. Evaluating our curated gene list, using a stringent posterior probability cutoff of >0.9, only 3 of the 19 GWAS hits colocalized with eQTLs (*CETP* in liver, h4: 0.985; *CETP* in adipose, h4: 0.983; *NPC1L1* in adipose, h4: 0.975; *PCSK9* in whole blood, h4: 0.960). Using a more lenient cutoff of >0.5, two more genomic regions showed evidence of colocalization (*APOE* in liver, h4: 0.873; *ANGPTL3* in liver, h4: 0.802; *NPC1L1* in liver, h4: 0.511), reflecting a colocalization rate of 26%. Surprisingly, other genes, such as the LDL receptor, did not show evidence of colocalization in the liver when these datasets were used (Figure 2). To improve power for the detection of eQTLs, we used MASH to update the summary statistics across tissues and re-ran coloc. No additional genes from our list showed evidence of colocalization. The results were also unchanged when using GWAS summary statistics without adjustment for coding variation. 

**Figure 3:**
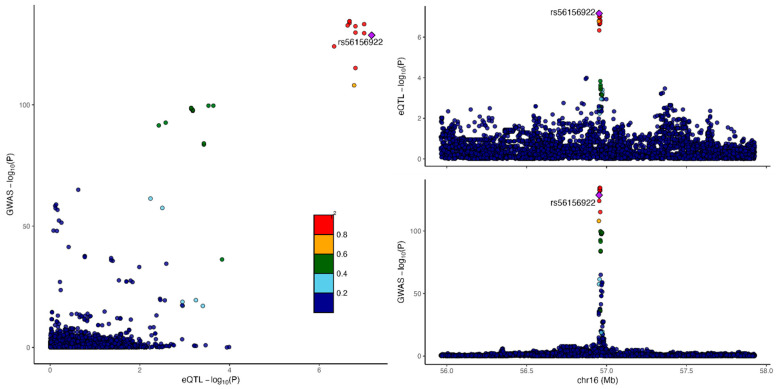
Example of successful eQTL and GWAS colocalization at CETP locus in adipose tissue, using summary statistics from European meta-analysis of LDL from [4], with GTEx v8. Here, the locus-level colocalization probability is 0.9823. The labeled SNP (purple) is the lead SNP, and other SNPs are color-coded according to their linkage disequilibrium r^2^ with the lead SNP. The left panel reflects the correlation between P_GWAS_ and P_eQTL_ for the analyzed variants. The top right and bottom right panels show Manhattan plots with apparent colocalization.

**Figure 4:**
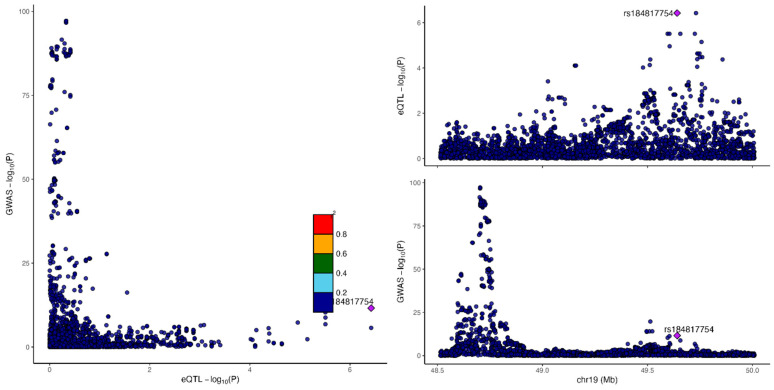
Example of lack of eQTL and GWAS colocalization at other LDL GWAS loci, such as at *FCGRT*, where there are highly significant GWAS associations with LDL (bottom right), highly significant eQTL associations (top right, lead eQTL variant in purple), overlapping SNPs between the GWAS and eQTL associations, but an LD structure that reflects different causative SNPs for each phenotype. Plot reflects summary statistics from European meta-analysis of LDL from [4], with GTEx v8 in liver. *FCGRT*, encoding the alpha-chain of the neonatal Fc receptor (FcRN), is a causal gene for hyperlipidemia. FcRN rescues albumin from degradation following endocytosis; decreased serum albumin leads to increased synthesis of lipoproteins including LDL [54].

We then applied a more recent colocalization method, fastEnloc, to three alternative fine-mapped GWAS results for LDL-C [4,27,28] from causalDB [29]. We used GTEx v8 statistics to evaluate for colocalization. When a stringent locus-level colocalization probability (LCP) of 0.9 was used, only three genes showed evidence of colocalization: *LPA* (liver), *CETP* (adipose) (Figure 3), and *ANGPTL3* (liver). With a relaxed posterior probability of 0.5, two additional genes showed evidence of colocalization, *APOB* (adipose) and *PCSK9* (adipose, whole blood) (Table 4). Despite a non-conservative approach with a low posterior probability cutoff, using multiple well-powered GWAS, and assessing multiple tissue types, only 5/19 genes (26%) showed evidence of colocalization and were present in our expert-curated list. Even though most genes on our expert-curated list had highly significant GWAS associations, highly significant eQTL associations, and overlapping SNPs between the GWAS and eQTL associations, there is a linkage disequilibrium structure that makes it clear that different SNPs are causative for the phenotype (Figure 4). 

## 4. Discussion

In this study, we identified known causal genes for LDL-C based on published literature demonstrating that the pharmacologic manipulation of the gene’s target led to increased or decreased LDL-C in humans. Some of these genes are also known Mendelian disease genes for hypercholesterolemia (for example, *APOB*, *APOE*, *LDLR*, *LPL*, and *PCSK9*), which adds further evidence beyond pharmacologic data that these genes are causal. Having identified a causal set of LDL-C genes, we show that there are eQTLs in relevant tissues that influence the expression of these genes in homeostatic conditions. However, our statistical colocalization approach demonstrates the failure of these eQTLs—which would be expected to influence LDL-C levels—to appear in GWAS. This reveals a missing regulation between genetic association and transcriptional abundance for causal LDL-C genes. 

Human genetic evidence has increasingly been reported as a predictor for the success of drugs in clinical trials [55], and consistent with this, we demonstrate that LDL-C GWAS hits are highly enriched for a set of known causal genes for hypercholesterolemia, over 527-fold compared to what is expected by chance. This validates the ability of GWAS to detect causal genes for disease states [55]. We also show that all causal hypercholesterolemia genes in our study have eQTLs; GTEx is highly powered to identify eQTLs, and studies have demonstrated that eQTL discovery in bulk, healthy adult tissue samples has achieved saturation [15]. Yet, for most LDL-GWAS hits, non-coding variants do not colocalize with known eQTLs for these causal LDL-C genes. In our study, only a minority of risk loci for causal LDL-C genes colocalized with eQTLs. This calls into question the hypothesis that genetic variants contribute to LDL-C by altering homeostatic gene expression. A limitation of our approach is that we focused on GWAS and eQTL studies performed in subjects of European ancestry. 

Our results extend the findings of a recent study that focused on a curated subset of 220 putative causal genes that harbored both GWAS-identified, common, non-coding variants as well as rare, coding genetic variation. Using this selected list of Mendelian genes, colocalization, transcriptome-wide association, and regulatory annotations nominated a candidate target for less than 10% of analyzed non-coding variants [14]. Serum LDL-C represents an ideal trait to extend the observation that non-coding GWAS risk variants may not impact the expression of a nearby putatively causal gene in healthy tissue. Multiple causal LDL-C genes have been identified, characterized, and therapeutically targeted, and the liver is a well-established relevant tissue type. The expression of these genes is not known to be developmentally regulated, so adult liver tissue eQTLs should be disease-relevant. LDL-C GWAS studies are well powered and have identified hundreds of loci that mediate LDL-C. An additional benefit is that our list of causal hypercholesterolemia genes is known to be dose-responsive with respect to target inhibition, such that changes in transcriptional abundance via eQTLs should be expected to impact the trait. 

There are several explanations for this “colocalization gap”. It has been argued that eQTLs, which are measured in bulk tissues in steady-state cellular conditions, may not reflect the specific cellular–environmental [56,57] or cell-type context [15,58,59] in which gene expression leads to disease. For example, it was recently found in a baboon model that hundreds of eQTLs emerge in adipose, liver, and muscle tissue after prolonged exposure to high dietary fat and cholesterol, with genomic localization that is distinct from steady-state eQTLs [60]. In fact, standard eQTL mapping in healthy tissue may be biased toward variants with minimal phenotypic effects, as these variants are better tolerated via natural selection [13]. Therefore, eQTLs in healthy tissue may predominately identify false positives, and dynamic eQTLs identified in disease-relevant contexts may identify true causal genes [61,62]. The notion that disease-relevant GWAS loci may have context-dependent regulatory effects not captured in most eQTL studies has implications for identifying new causal genes and drug targets from other cardiometabolic GWAS studies.

A possibility is that disease-relevant eQTLs are limited to rare cell populations. Within the liver, functionally specialized hepatocytes are sequentially organized into periportal, mid-zonal, and perivenular zones along the sinusoidal tubules of the liver cell plate within liver lobules; recent studies using gene expression patterns, flow cytometry, and immunohistochemical examinations have identified 20 discrete cell populations of hepatocytes [63]. The periportal zone contains hepatocytes that express high levels of HMGCoA reductase, the key enzyme involved in endogenous cholesterol biosynthesis, and secrete and endocytose apolipoprotein particles, including *APOE* [64]. It remains to be seen whether analyzing eQTLs from rare subpopulations or single-cell eQTLs that may be underrepresented in bulk tissue could improve colocalization. 

We suggest that alternative approaches may be required to address the complexities of assigning target genes to GWAS-identified variants. This includes case-control eQTL cohorts to identify disease-specific eQTLs, increases in sample size and ancestral diversity, the analysis of stimulated conditions or cellular contexts that are only accessible via single-cell RNA sequencing, the analysis of the impact of genetic variation on mechanisms other than gene expression (protein expression, splicing, methylation, etc.), and emerging experimental techniques using CRISPR-based variant editing or the CRISPR perturbation of enhancers. 

## 5. Conclusions

Here, we provide evidence that eQTLs do not effectively link GWAS variants to causal genes. We specifically challenge the assumption that non-coding variants affect circulating lipid levels by impacting the baseline expression of causal LDL-C-related genes. These results highlight the complexity of genetic regulatory effects for causal hypercholesterolemia genes.

## Figures and Tables

**Table 1:** Validated hypercholesterolemia disease genes as proven via pharmacological manipulation in humans. Gene of drug target, drug examples, and published literature demonstrating dose-dependent effect of target modulation on LDL-C are provided.

Gene	Drug Examples	Evidence of Clinically Meaningful, Statistically Significant, Dose-Dependent Effects of Target Modulation on LDL-C in Humans
HMGCR	Lovastatin Simvastatin Atorvastatin Pravastatin	Log-linear dose response for LDL-C across range of defined daily doses; “rule of 6%” describes additional % reduction in LDL-C from pretreatment for each statin dose doubling [30].
LDLR	Statins RGX-501	Decreases in liver cholesterol via statin inhibition of HMG CoA reductase activates SREBP processing, increasing the number of LDL receptors displayed on liver cell membranes [31].
ANGPTL3	ARO-ANG3 Evinacumab Vupanosen	Dose-dependent reductions of mean ANGPTL3 (between 62–92%) and LDL-C reductions (between 23% and 37%) in patients with hypercholesterolemia [32].
THRB	Resmetirom Sobetirome Eprotirome VK2809	Dose-dependent reductions of LDL-C up to 18.8% in heterozygous familial hypercholesterolemia [33].
ACLY	Bempedoic acid	Dose-dependent LDL-lowering effect in patients with hypercholesterolemia up to 26.6% [34].
NPC1L1	Ezetimibe Canosimibe KT6-971	Dose-dependent LDL-C lowering up to 19.4% as monotherapy in hypercholesterolemia [35].
CETP	Obicetrapib Evacetrapib Anacetrapib JTT-302	Dose-dependent LDL cholesterol lowering up to 45.3% [36].
APOC3	ARO-APOC3 Olezarsen Volanesorsen RBD-5044	Dose-dependent reductions in LDL-C and ApoB up to 19% [37].
LPA	AMG890 Peclacarsen Zerlasiran LY3819469	Dose-dependent reduction in low-density lipoprotein cholesterol up to 26% [38].
PCSK9	Inclisiran Alirocumab Evolocumab MK-0616 AZD-8233 AZD-6615 LIB-003 Bococizumab LY-3015014 LDL-4 × 4	Dose-dependent reductions in LDL-C up to 50% [39].
MTTP	Lomitapide SLx-4090	Dose-dependent LDL-C reductions up to 65% [40].
FGF21	Pegozafermin Efruxifermin	Reductions in LDL-C, non-HDL-C, and apolipoprotein B up to 11.1% from baseline [41].
PPARA	Fenofibrate Gemcabene Gemfibrozil AVE-8134	LDL-C reductions ranging from 5% to 35% [42].
FASN	Denifanstat	Dose-dependent reductions in LDL-C up to 18.7% [43].
APOB	Mipomersen SPC4955	Dose-dependent lipid-lowering effects, up to 24.7% [44].
LXR/NR1H3	BMS-85927 TLC-2716	Dose-dependent increases in LDL-C with LXR agonism in both healthy subjects and in dyslipidemic patients on statin therapy [45]; dose-dependent decreases in LDL-C with LXR inverse agonist [46].
PPARD	Seladelpar	Dose-dependent reductions in LDL-C up to 21.8% [47].
APOE	AEM-28	Dose-dependent VLDL-C reductions up to 79.2% in Phase 1 [48]; in primates, LDL-C reduced up to 64% [49].
LPL	Alipogene tiparvovec	Decreases in plasma triglyceride levels for up to 26 weeks with sustained decrease in chylomicron triglyceride levels [50].
CYP7A1	NGM282 Chenodeoxycholic acid Obeticholic acid	Inhibitors of this rate-limiting enzyme in the conversion of cholesterol to bile acids increase plasma LDL-C dose-dependently up to 50% [51,52].
FCGRT	Batoclimab	Dose-dependent increases in serum cholesterol up to 58.9% [53].

**Table 2:** Validated hypercholesterolemia genes, presence of a representative tissue eQTL (GTEx eQTL with smallest *p*-value), LDL-C GWAS locus from largest meta-analysis of European ancestry-specific GWAS of LDL, lead SNP, and GWAS *p*-value. Almost all genes in our set are the closest gene to a significant LDL-C genomic locus. Almost all genes in our set have a significant eQTL in a disease-relevant tissue.

Gene	eQTL *p*-Value (Tissue)	GWAS Locus	SNP	GWAS *p*-Value
HMGCR	4.4 × 10^−7^, adipose	5:74632154–74657929	rs12916	1.10 × 10^−20^
LDLR	1.7 × 10^−8^, whole blood	19:11200038–11244492	rs73015024	1.34 × 10^−1575^
ANGPTL3	1.1 × 10^−5^, liver	1:63063158–63071830	rs147371901	6.48 × 10^−174^
THRB	4.3 × 10^−6^, liver	3:24158651–24536773	rs12638970	5.04 × 10^−33^
ACLY	3.0 × 10^−7^, adipose	17:40023161–40086795	rs72836561	1.25 × 10^−201^
NPC1L1	1.7 × 10^−18^, adipose	7:44552134–44580914	rs148825701	2.03 × 10^−126^
CETP	6.9 × 10^−10^, ileum	16:56995762–57017757	rs6499863	3.14 × 10^−135^
APOC3	2.4 × 10^−5^, adipose	11:116700422–116703788	rs139524394	2.86 × 10^−191^
LPA	7.4 × 10^−9^, liver	6:160952515–161087407	rs10455872	3.86 × 10^−282^
PCSK9	2.3 × 10^−28^, adipose	1:55505221–55530525	rs11591147	7.23 × 10^−1039^
FGF21	1.4 × 10^−4^, adipose	N/A	N/A	N/A
PPARA	2.0 × 10^−5^, adipose	22:46546424–46639653	rs6008798	5.88 × 10^−13^
MTTP	2.0 × 10^−5^, adipose	4:100484918–100545156	rs28497720	4.3 × 10^−28^
FASN	5.3 × 10^−14^, whole blood	N/A	N/A	N/A
APOB	4.9 × 10^−29^, adipose	2:21224301–21266945	rs72654423	1.8 × 10^−693^
LXR/NR1H3	3.2 × 10^−7^, whole blood	11:47269851–47290396	rs61882680	1.01 × 10^−11^
PPARD	3.0 × 10^−5^, liver	6:35310335–35395968	rs73413718	7.00 × 10^−9^
APOE	4.7 × 10^−5^, whole blood	19:45409011–45412650	rs75687619	3.82 × 10^−6283^
LPL	8.5 × 10^−23^, whole blood	8:19709228–19874770	rs769111033	6.39 × 10^−9^
CYP7A1	8.4 × 10^−9^, adipose	8:59402737–59412795	rs9297994	1.94 × 10^−97^
FCGRT	9.5 × 10^−17^, adipose	19:49960073–50079685	rs142385484	1.16 × 10^−15^

**Table 3:** LDL-C GWAS loci are enriched for pharmacologically validated hypercholesterolemia targets. In total, the LDL GWAS identified 402 significant genomic loci. Of the 21 expert-curated and validated genes implicated in LDL-C via pharmacologic validation, 19 genes were the closest gene to a significant LDL-C genomic locus. This corresponds to a 527-fold enrichment compared to what is expected by chance (95% confidence interval: 126 to 5376; Fischer’s exact *p* < 2.2 × 10^−16^).

	Validated Hypercholesterolemia Gene (*n* = 21)	Not a Validated Hypercholesterolemia Gene (*n* = 21,673)
LDL GWAS risk loci gene (*n* = 402)	19	383
Not LDL GWAS risk loci gene (*n* = 21,292)	2	21,290

**Table 4:** Results are shown for loci that have positive colocalization results using FastenLoc with locus-level colocalization probability (LCP) > 0.5. Gene, tissue of eQTL, LDL GWAS study, number of fine-mapped SNPs in locus, LCP, lead variant, reference/alternate allele, distance to transcription start site (TSS) for lead variant, minor allele frequency (MAF) and GTEx *p*-value for lead variant are also shown.

Gene	Tissue	GWAS Study	Fine-Mapped SNPs (*n*)	LCP	Lead Variant	Reference: Alternate	TSS Distance	MAF	GTEX Nominal *p*-Value
LPA	Liver	Graham	46	0.9989	chr6_160611297	G:C	−52,962	38%	7.3 × 10^−6^
CETP	Adipose	Graham	12	0.9823	chr16_56957451	C:T	−4399	34%	2.1 × 10^−7^
CETP	Adipose	Teslovich	12	0.9418	chr16_56955678	C:T	−6172	34%	2.3 × 10^−7^
ANGPTL3	Liver	Graham	168	0.9408	chr1_62604866	AGTTAATGTG:A	7379	35%	3.4 × 10^−4^
ANGPTL3	Liver	Teslovich	168	0.8111	chr1_62584148	G:A	−13,339	34%	2.8 × 10^−4^
APOB	Adipose	Kettunen	28	0.7413	chr2_21067195	C:T	23,122	20%	6.0 × 10^−1^
APOB	Adipose	Teslovich	28	0.7358	chr2_21065354	G:A	21,281	20%	6.4 × 10^−1^
PCSK9	Adipose	Teslovich	8	0.7184	chr1_55030366	T:C	−9182	19%	7.2 × 10^−4^
PCSK9	Blood	Kettunen	5	0.7068	chr1_55052794	A:G	13,246	41%	2.8 × 10^−10^
PCSK9	Adipose	Kettunen	8	0.7049	chr1_55021673	C:G	−17,875	24%	7.2 × 10^−4^
CETP	Liver	Graham	12	0.6467	chr16_56957451	C:T	−4399	36%	2.1 × 10^−3^
CETP	Liver	Teslovich	12	0.6197	chr16_56959412	C:A	−2438	36%	2.1 × 10^−3^

## Data Availability

The original contributions presented in this study are included in the article. Further inquiries can be directed to the corresponding author.
